# Fabrication of a Chalcogenide Glass Microlens Array for Infrared Laser Beam Homogenization

**DOI:** 10.3390/ma14205952

**Published:** 2021-10-10

**Authors:** Fan Zhang, Qing Yang, Hao Bian, Shaokun Wang, Minjing Li, Xun Hou, Feng Chen

**Affiliations:** 1 State Key Laboratory for Manufacturing System Engineering and Shaanxi Key Laboratory of Photonics Technology for Information, School of Electronic Science and Engineering, Xi’an Jiaotong University, Xi’an 710049, China; zf19900625@stu.xjtu.edu.cn (F.Z.); wang_shao_kun@163.com (S.W.); houxun@mail.xjtu.edu.cn (X.H.); 2 School of Mechanical Engineering, Xi’an Jiaotong University, Xi’an 710049, China; yangqing@mail.xjtu.edu.cn (Q.Y.); liminjing92@stu.xjtu.edu.cn (M.L.); 3 Wuhan National Laboratory for Optoelectronics, Huazhong University of Science and Technology, Wuhan 430074, China

**Keywords:** chalcogenide glass, infrared microlens, IR laser homogenizer

## Abstract

Infrared (IR) microlens arrays (MLA) have attracted increasing interest for use in infrared micro-optical devices and systems. However, the beam homogenization of IR laser light is relatively difficult to achieve because most materials absorb strongly in the IR wavelength band. In this paper, we present a new method for the application of double-sided quasi-periodic chalcogenide glass (ChG) MLAs to infrared laser homogenization systems. These are non-regular arrays of closely spaced MLAs. The double-sided MLAs were successfully prepared on the ChG surface using a single-pulse femtosecond laser-assisted chemical etching technique and a precision glass molding technique. More than two million close-packed microlenses on the ChG surface were successfully fabricated within 200 min. By taking advantage of ChG’s good optical performance and transmittance (60%) in the infrared wavelength band (1~11 μm), the homogenization of the IR beam was successfully achieved using the ChG quasi-periodic MLA.

## 1. Introduction

With the development of infrared technology, infrared lasers have been gradually applied in various fields, such as atmospheric monitoring [1], remote sensing [2], night vision systems [3], IR countermeasures [4], and medical applications [5]. However, many applications of IR lasers, such as laser manufacturing [6], high-performance illumination [7,8], and laser diagnostics [9], require a homogeneous intensity distribution of the laser beam over its complete profile. Various methods can be used to realize laser beam homogenization, such as diffractive optics, aspherical lens systems, polygonal light tubes, liquid crystal spatial modulators, and MLAs [10,11,12,13,14]. With advantages such as their simple structure, low transmission loss, high damage threshold, and low requirements for incident light intensity distribution, MLAs are widely used optical elements for laser beam homogenization [15,16,17]. MLAs belong to a multi-aperture beam integration system. MLAs can break up a non-homogeneous laser into many beams, and each beam can be superimposed onto the microdisplay with the help of an additional lens. As a result, MLA beam homogenizers exhibit excellent optical performance. However, MLAs consisting of periodically distributed microlenses produce interference patterns, especially when highly coherent light is used. A solution is proposed to reduce the interference fringe contrast by perturbing the spatial distribution of the microlenses using a double-sided non-periodically arranged MLA.

Booming applications in the IR region have created a demand for low-cost, highly efficient IR optics, but there have been few studies on infrared optics. Specifically, most materials have strong absorption in the infrared band, so the homogenization of IR lasers is more difficult [18]. Chalcogenide glass is an excellent IR material, whose constituent elements mainly include the three sulfur group elements—sulfur (S), selenium (Se), and tellurium (Te)—and group IIIA-VA elements—germanium (Ge), gallium (Ga), arsenic (As), and antimony (Sb) [19,20,21]. ChG has excellent optical properties, specifically: (1) a transparent region covering 1.064 μm lasers and three infrared atmospheric windows of 1~3 μm, 3~5 μm, and 8~12 μm; (2) a low refractive index temperature coefficient and a low dispersion; and (3) high optical uniformity. Since its introduction in the 1950s, ChG has been widely used in infrared lenses [22,23], fiber-optic lasers [24], guided-wave photonic devices [25], and phase-change materials [26]. Therefore, chalcogenide glass with a microlens array can be used to homogenize infrared beams. In general, the commonly used method for processing ChG is single-point diamond turning (SPDT), which is time-consuming and only suitable for ordinary-sized lenses. In addition, the complexity and high cost of the procedure also limit its practical applications. Due to the low transition temperature (Tg) and stable physicochemical properties of ChG, a precision glass molding process is considered the most desirable process for processing ChG, which can be precisely molded into its final shape in a single operation by providing heat and pressure without affecting major changes to the internal structure [27]. For high-volume production, precision glass forming is preferable to traditional methods, as the forming time is much shorter than single-point diamond turning and polishing processes, and the mold can be reused, making the cost much lower [28,29]. Nevertheless, the use of a high-quality master mold with the needed features and functions of the target IR components for ChG is critical in the precision glass molding process.

Herein, an ultra-smooth homogenizer based on the ChG double-sided MLA was proposed for shaping an infrared pulse laser beam with a flat-topped profile intensity distribution. The technology is based on a single-pulse femtosecond laser-assisted chemical etching and precision glass molding process to achieve the rapid preparation of the double-sided MLA homogenizer. Within 180 min, about one million close-packed ChG microlenses with quasi-periodic distribution could be fabricated. Due to the high transmittance of ChG in the infrared band, we carried out the homogenization of laser beams with a wavelength of 1064 nm using the ChG double-sided MLA homogenizer. The results demonstrate the potential application of the fabricated double-sided ChG MLA for IR laser homogenization.

## 2. Methods and Experimental Procedure

**Figure 1:**
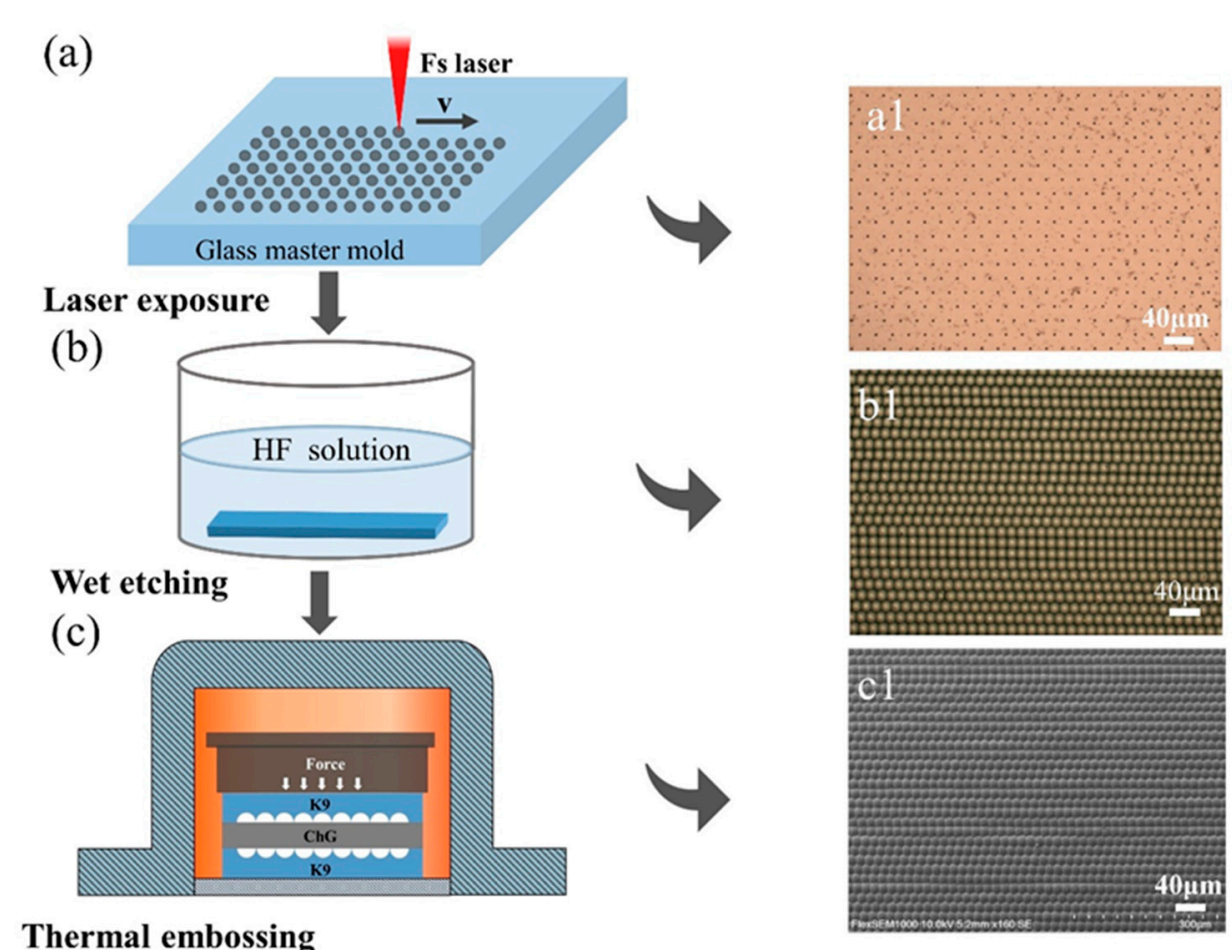
Schematic of the fabrication process. (**a**) Laser-induced BK7 glass modification; (**a1**) an optical microscope image of the laser-modified area; (**b**) concave microstructures with smooth surfaces were formed by the chemical wet etching process; (**b1**) an optical microscope image of the irregular concave microstructures; (**c**) the ChG quasi-periodic MLAs were fabricated through a precision molding process; (**c1**) SEM images of the ChG quasi-periodic MLAs.

The ChG double-sided MLAs were fabricated in two steps, including a single-pulse femtosecond laser-assisted chemical etching and precision glass molding process, as shown in Figure 1. The mold can be used thousands of times without the need for repair, so the ChG MLA is manufactured starting with a hardened concave mold, which is made on a BK7 optical glass substrate by means of the efficient single-pulse femtosecond laser-assisted chemical etching technology [30,31]. An 800 nm femtosecond laser beam with a repetition rate of 1 kHz and a pulse width of 50 fs was focused by an objective lens (Nikon, Tokyo, Japan, NA = 0.5). A series of ablation craters in a v μm separation space was formed by continuous scanning at v mm/s (experimental conditions of 10 ≤ v < 40) and at a laser repetition frequency of 1 kHz (Figure 1(a,a1)). Then, the BK7 glass was ultrasonically cleaned in deionized water to remove the laser ablation ejecta. The irradiated BK7 glass was then treated with an 8% (*w*/*v*) hydrofluoric acid solution (HF) in an ultrasonic water bath (Figure 1b). After about 30 min of etching, a quasi-periodic arrangement of MLAs was produced on the surface of the BK7 glass (Figure 1(b1)). 

Using precision glass molding, the double-sided IR MLA was replicated using two quasi-periodic templates. A pre-dried piece of ChG was sandwiched between the two quasi-periodic templates, with the structured side of the template touching the ChG. The assembled module was then mounted in a high-temperature oven, as shown in Figure 1c. The ChG used in this study was NBU-1R1 (Ge_20_Sb_15_Se_65_. NingboUniv., Ningbo, China). The physical properties of the ChG are shown in Table 1.

**Figure 2:**
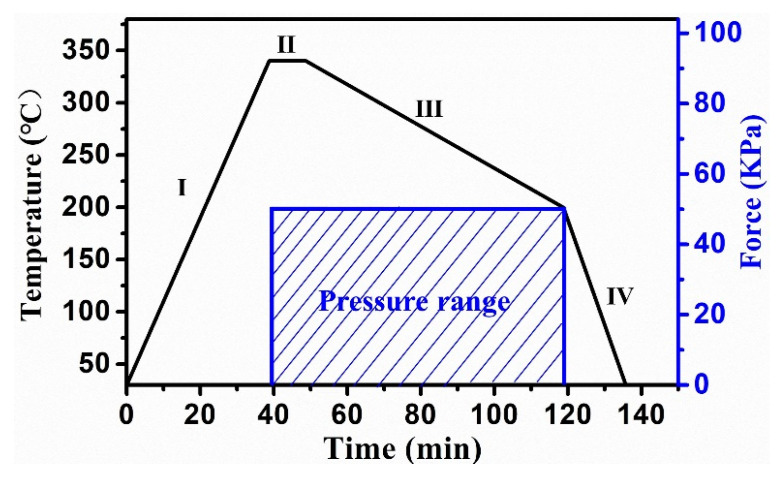
Schematic diagram of the molding process. I: heating; II: pressing; III: slow cooling; IV: rapid cooling.

The molding process was divided into four stages, as shown in Figure 2: a heating stage, a pressing stage, a slow cooling stage, and a rapid cooling stage [32]. At each stage, the molding process was carried out as follows: (I) the mold and the ChG were heated to the molding temperature in a high-temperature furnace; (II) the heated glass was pressed between heavy blocks; (III) the pressed ChG was slowly cooled to release the thermal stresses induced in the ChG; (IV) when the thermal stresses inside of the ChG had been fully released, the ChG began to cool rapidly to room temperature. The surface morphology and main profile of the molded microlens were measured using a scanning electronic microscope (SEM) (FlexSEM 1000, Hitachi Corporation, Tokyo, Japan) and a laser scanning confocal microscope (LSCM) (OLS4000, Olympus Corporation, Tokyo, Japan).

## 3. Results and Discussion

**Figure 3:**
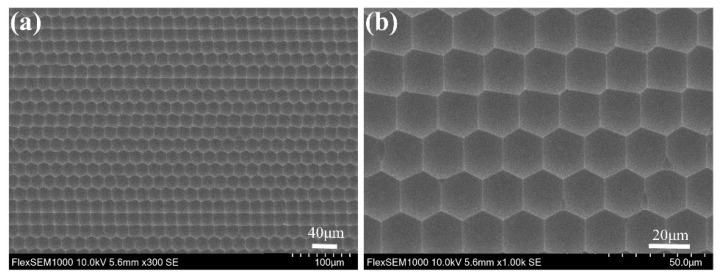
(**a**) Top-view SEM image of the quasi-periodic MLA and (**b**) a magnified SEM image of the quasi-periodic MLA.

By setting the scanning speed to 20 mm/s and the laser power to 5 mW, the quasi-periodic microlenses could be observed, showing an average size of 20 μm and a total area easily covering several square inches. Figure 3 shows the SEM image of the MLA. The magnified image of the quasi-periodic MLA in Figure 3 clearly shows that the prepared MLA along the Y-axis has an irregular shape, a smooth surface, and a high filling factor (up to 100%).

**Figure 4:**
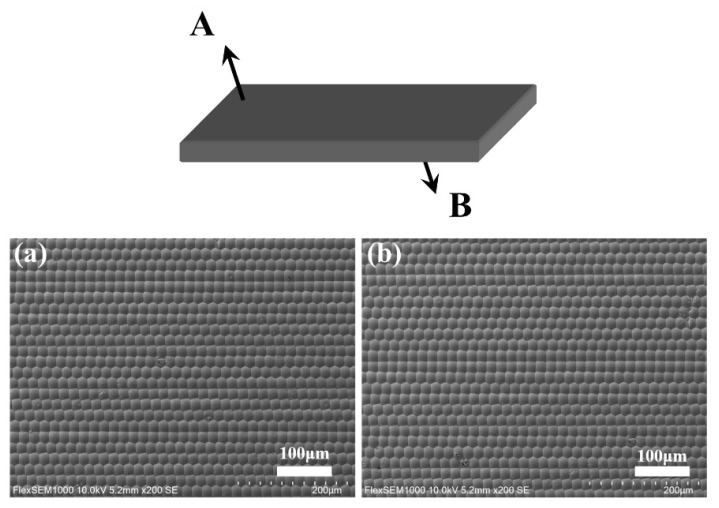
(**a**) Top-view (A-side of a double-sided MLA) SEM image of the quasi-periodic ChG MLA. (**b**) SEM image of the B-side of the quasi-periodic ChG MLA.

The molding conditions were determined from a previous study, which examined the effect of temperature and pressure on the plasticity of ChG, as shown in Table 2 [22]. To prevent the ChG from breaking, a slow cooling process followed by a rapid cooling process was chosen for the cooling stage. The molding load was maintained at 50 kPa for the pressing and slow cooling stages. Figure 4 shows the SEM images of the A and B surfaces of the ChG glass under the given molding conditions. It can be seen from the figure that there are no bubbles or defects on the surface of the ChG microlenses, and there is no MLA breakage or glass adhesion to the mold surface. The fill factor of the ChG microstructures reached 100%.

**Figure 5:**
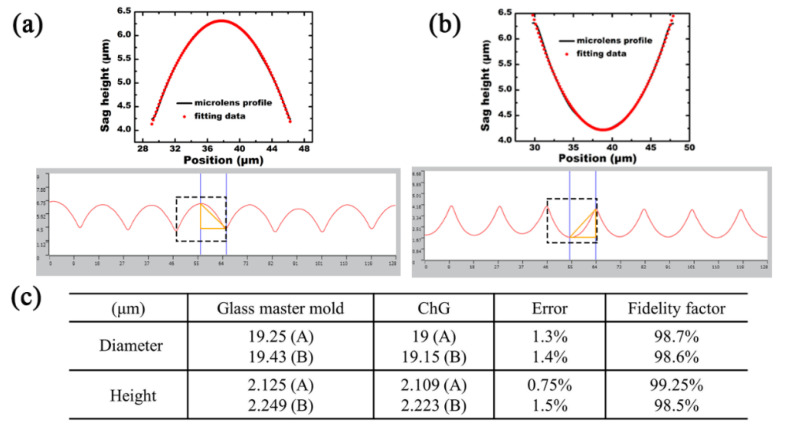
(**a**) The cross-section of the convex MLAs; (**b**) the cross-section of the concave MLAs; insets in (**a**,**b**) are the enlarged view of the data in the box and its profile fitting; (**c**) the fidelity factor of the convex MLAs.

To characterize the cross-section of the prepared microstructures, laser scanning confocal microscopy was utilized. The microstructures were quasi-periodically arranged irregular microlenses with a size of 2 cm × 2 cm covering the whole area of the ChG. The profiles of the BK7 MLA and the ChG MLA are shown in Figure 5, respectively. To verify the uniformity of the microlenses, different areas of the ChG were measured, and the data were statistically processed and analyzed. The results show that the average diameter and sag height of the microlenses were 19 μm and 2.109 μm, respectively. In addition, according to measured and theoretical fitting data, the microlenses had a parabolic shape, as shown in the insets of Figure 5a,b. The focal length of the microlens can be calculated according to f = D^2^/8h (n − 1), where D and h are the diameter and sag height of the microlens, respectively, and n is the reflectivity of the material. According to the formula, the focal lengths of the ChG microlenses and the BK7 MLA were 13.4 μm and 43.59 μm, respectively. The deformation ratio was ≈1% (Figure 5c), demonstrating that this simple method could realize quasi-periodic MLAs on the surface of ChG with relatively high fidelity.

**Figure 6:**
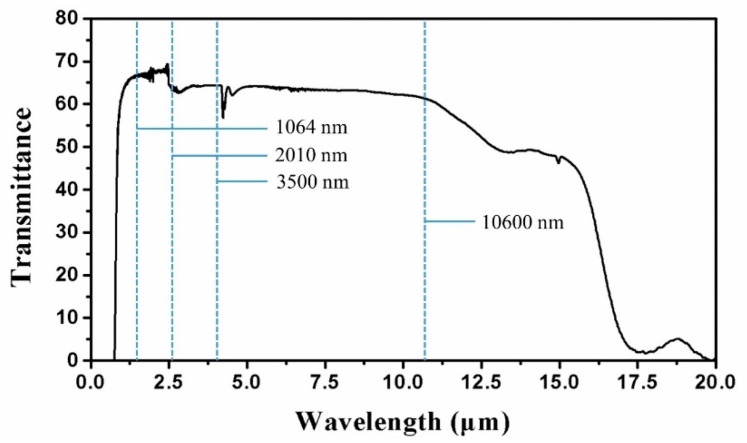
Measured transmittance of the ChG MLA.

As an infrared optics device, transparency in the infrared band and isolation in the visible band are key features for practical applications. The transmittance of the ChG MLA was measured by a Fourier infrared spectrometer (Nicolet iS10, Thermo Fisher Corporation, Waltham, MA, USA). The feature size of the ChG MLA fabricated was around 20 μm, so the scattering of wavelengths is expected at such a scale. The loss arose with wavelengths beyond 20 μm, as shown in Figure 6. For a spectrum smaller than the feature size of the ChG MLA, the scattering is not obvious. In addition, if the distance between the infrared device and the detector of the Fourier infrared spectrometer is relatively short, the energy loss is relatively small. So, within this spectrum range, there is no severe loss of beam energy. Figure 6 shows that there is a sharp cut-off at the edge of the visible spectrum, and the ChG MLA (θ = 0°) shows a relatively high transmittance throughout the IR region, especially in some important IR spectroscopic technology windows, such as 1064 nm for Nd: YAG lasers; 2010 nm for Tm: YAG lasers; 3500 nm for Cr: CdSe lasers; 10,600 nm for CO_2_ lasers; and so on. This remarkable spectral selectivity bodes well for its wide application in the IR region.

**Figure 7:**
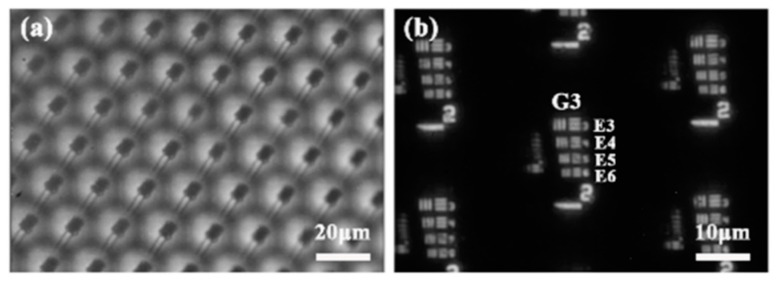
(**a**) Clear IR images obtained from the ChG MLAs; (**b**) IR images of a standard USAF1951 resolution target plate sampled by the MLA.

The infrared imaging quality is the most basic optical characteristic of infrared microlenses. The infrared imaging capability of the quasi-periodic MLA was tested using an infrared microscope system, and the test result is shown in Figure 7a. From the figure, it can be seen that the prepared ChG quasi-periodic microlenses had good imaging performance and could form an image with clear edges. The imaging of the device was also measured quantitatively using a standard resolution plate (USAF 1951), and it can be seen in Figure 7b that clear imaging in both horizontal and vertical directions was achieved for three group 6 units, corresponding to a resolution of 14.3 lp/mm [33,34]. The structure of this experiment provides new ideas toward the realization of a high-quality and inexpensive infrared imaging device, compared to expensive imaging devices in the mid- and far-infrared bands.

**Figure 8:**
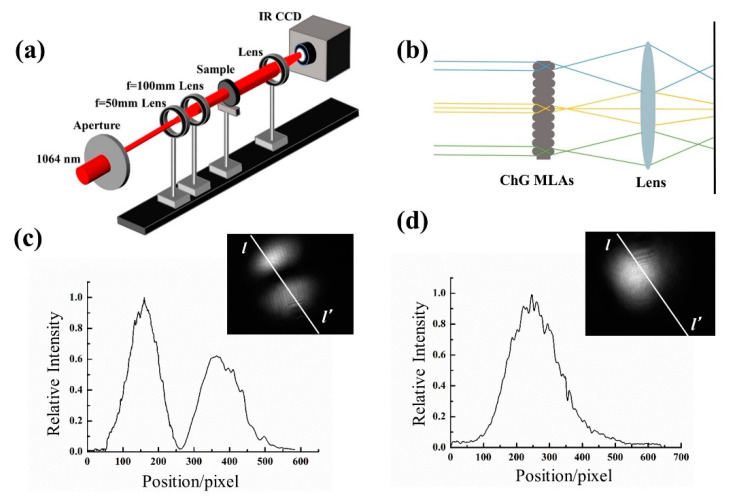
(**a**) Optical system used for measuring the illumination distribution; (**b**) a principle diagram of light beam homogenization by the microlens array; (**c**,**d**) cross-sections of light intensity before the homogenization of infrared light (multimode and Gaussian beam).

Beam homogenization is one of the most common applications for microlens arrays. Most microlens arrays are fabricated on polymer or plain glass, which are usually used for visible wavelengths. With a high transmittance for wavelengths, ranging from 1 to 11 µm, the ChG MLAs can be used for IR beam homogenization. The light beam homogenization was measured experimentally using the setup shown in Figure 8a. The light source was a semiconductor multimode laser with a wavelength of 1064 nm, and the output pattern was captured by an infrared CCD camera. As shown in Figure 8b, when the light passed through the ChG double-sided quasi-periodic MLA, the wavefront of the incident beam was divided into a series of wavelets, with each focal point of the microlens being a separate light source. The wavelets were then superimposed onto the screen by focusing, resulting in a uniform distribution of light intensity. The laser emitted a beam with a diameter of about 1 mm, and the incident laser was expanded to increase its diameter twice in order to allow the laser to cover more microlens units. The 1064 nm infrared semiconductor laser, which emits a multimode laser pattern as shown in the inset of Figure 8c, was shaped by the aperture and transformed into a single Gaussian beam (inset of Figure 8d). 

**Figure 9:**
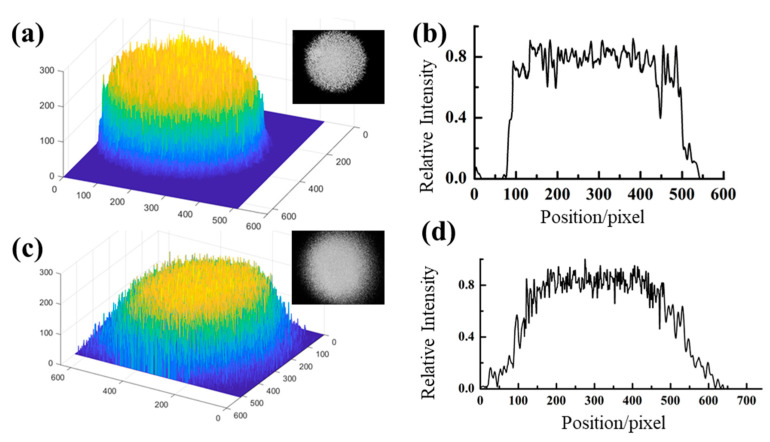
3D images (**a**,**c**) and cross-sections (**b**,**d**) of light intensity after the homogenization of the high-fluence pulsed femtosecond laser beam infrared light (multimode and Gaussian beam). Inset images are the illumination distribution of the laser beam after homogenization.

After the homogenization of the ChG microlens array, the intensity distribution of the infrared laser in both modes spread to a flat-top distribution, shown in Figure 9. The infrared light beam (1064 nm) was significantly homogenized by the ChG MLAs compared to the incident light. Moreover, digital manipulation was utilized to analyze the illumination distribution of the laser beam that was detected. Figure 9a,c show the intensity distribution of the homogenized beam. Meanwhile, the normalized intensity at the central horizontal line of the output pattern is shown in Figure 9b,d. The light patterns of the diffuser with different rotation parameters were compared. In general, the homogeneous light spot was essentially constant with an increasing rotation angle. We also measured the transmittance of the double-sided MLAs at 1064 nm. The results show that the transmittance of the double-sided MLAs was around 62%, independent of the scanning angle.

## 4. Conclusions

In summary, a large area of closely-packed, uniform double-sided quasi-periodic ChG MLAs were prepared using femtosecond laser wet etching combined with a precision molding technique and successfully applied to achieve the homogenization of infrared lasers. For mass production, this method shows great potential for preparing quasi-periodic closely-packed infrared microlenses. The focusing and imaging characteristics were tested, and the results showed that the ChG MLA has good infrared optical properties. Based on the good transmittance of the ChG MLA in the infrared band of 1~11 μm, the distribution of a 1064 nm semiconductor laser was homogenized from a Gaussian to a flat-top distribution. All the evidence proves that this large-scale infrared quasi-periodic MLA can be widely used in fields such as the night illumination of infrared lasers and infrared laser processing.

## Figures and Tables

**Table 1:** The physical properties of the chalcogenide glass (NBU-IR1).

Physical Properties	Value
Thermal conductivity (Wm^−1^K^−1^)	0.23
Thermal expansion coefficient (K^−1^)	1.41 × 10^−5^
Transition temperature (T_g_) (°C)	284
Refractive index at 10 μm	2.59609 (25 °C), 2.58928 (80 °C)
Thermo-optic coefficient at 10 μm (K^−1^)	5.8 × 10^−5^
Young’s modulus (GPa)	19.11

**Table 2:** The molding conditions and process parameters used in this study.

Stage	Heating	Pressing	Slow Cooling	Rapid Cooling
Temperature (°C)	340	340	200	20
Pressing force (kPa)	—	50	50	—
Process time (min)	40	10	70	18

## Data Availability

Not applicable.
